# Early detection of sensorineural hearing loss in Muckle-Wells-syndrome

**DOI:** 10.1186/s12969-015-0041-9

**Published:** 2015-11-04

**Authors:** Jasmin B. Kuemmerle-Deschner, Assen Koitschev, Pascal N. Tyrrell, Stefan K. Plontke, Norbert Deschner, Sandra Hansmann, Katharina Ummenhofer, Peter Lohse, Christiane Koitschev, Susanne M. Benseler

**Affiliations:** Department of Pediatrics, Division of Pediatric Rheumatology, University Hospital Tuebingen, Hoppe-Seyler-Str. 1, D-72076 Tuebingen, Germany; Department of Otorhinolaryngology, Head and Neck Surgery, Klinikum Stuttgart, Stuttgart, Germany; Department of Medical Imaging, University of Toronto, Toronto, Canada; Department of Otorhinolaryngology, Head and Neck Surgery, University Hospital Halle (Saale), Halle (Saale), Germany; Department of Anesthesiology and Intensive Care Medicine, University Hospital Tuebingen, Tuebingen, Germany; CeGaT, Center for Genomics and Transcriptomics, Tuebingen, Germany; Rheumatology, Department of Pediatrics, Alberta Children’s Hospital, University of Calgary, Calgary, Canada

**Keywords:** *NLRP3* mutation, Muckle-Wells-Syndrome, Cryopyrin-associated periodic syndrome, Autoinflammatory syndromes, Hearing loss, Inner ear, Pure tone average

## Abstract

**Background:**

Muckle-Wells-syndrome (MWS) is an autoinflammatory disease characterized by systemic and organ-specific inflammation due to excessive interleukin (IL)-1 release. Inner ear inflammation results in irreversible sensorineural hearing loss, if untreated. Early recognition and therapy may prevent deafness. The aims of the study were to characterize the spectrum of hearing loss, optimize the otologic assessment for early disease and determine responsiveness to anti-IL-1-therapy regarding hearing.

**Methods:**

A single center prospective cohort study of children and adults with MWS was performed. Standardized clinical, laboratory and otologic assessments including standard pure tone audiometry, additional high tone thresholds, vestibular organ testing, tinnitus evaluation and functional disability classes were determined serially. Pure-tone-average models were developed and evaluated. Risk factors for hearing loss and the impact of anti-IL-1 treatment were determined.

**Results:**

A total of 23 patients with genetically confirmed MWS were included, of whom 63 % were females; 52 % were children. At baseline all patients had active MWS; 91 % reported clinically impaired hearing with 74 % having an abnormal standard assessment (0.5–4 kHz). In contrast, high frequency pure tone averages (HF-PTA) were abnormal in all symptomatic patients including those with early hearing loss (sensitivity 100 %). Females were at highest risk for hearing loss even after adjustment for age (*p* = 0.008). Treatment with IL-1 blockade resulted in improved or stable hearing in 91 % of patients.

**Conclusions:**

Early inner ear inflammation in MWS primarily affects the high frequencies, beyond the range of standard otologic assessment tools. The HF-PTA is a sensitive tool to detect imminent hearing loss and monitor treatment response.

**Electronic supplementary material:**

The online version of this article (doi:10.1186/s12969-015-0041-9) contains supplementary material, which is available to authorized users.

## Background

Muckle-Wells syndrome (MWS) is an autosomal dominant autoinflammatory disease in the clinical spectrum of cryopyrin-associated periodic syndrome (CAPS). CAPS comprise the mildest form, familial cold autoinflammatory syndrome (FCAS), the intermediate MWS and the most severe phenotype chronic infantile neurological cutaneous and articular syndrome (CINCA) or neonatal-onset multisystem inflammatory disease (NOMID) [1]. First described in 1962, MWS was characterized by the triad of urticaria, deafness and reactive amyloid A (AA) amyloidosis [2]. In 2001, Hoffman et al., reported gain-of-function mutations in the *NLRP3-*gene *(CIAS1)* on chromosome 1q44 encoding the protein NLRP3 (cryopyrin) in MWS [3–5]. Subsequently NLRP3/cryopyrin was identified to be a key protein of the multiprotein cytoplasmic complex named inflammasome [6]. In CAPS patients, impaired NLRP3/cryopyrin results in excessive release of the active form of interleukin (IL)-1β [7], causing severe inflammatory symptoms including fever, rash, conjunctivitis, headache, arthralgia/arthritis and fatigue [8]. Devastating organ disease of MWS includes amyloidosis and deafness [9].

Sensorineural hearing loss in MWS often rapidly progresses from mild high-tone deficits to complete deafness [10, 11]. Early hearing loss primarily affects high frequencies of  ≥ 6 kHz reflecting the characteristic high sensitivity pattern of hair cells to injury as described in other systemic conditions such as rheumatoid arthritis and diabetes [12, 13]. Goldbach-Mansky and co-workers were able to visualize the inflammatory injury in CAPS on MRI studies [14, 15]. Early inner ear inflammation and hearing loss may initially not impact communication in quiet. Reports suggest the reversibility of early inner ear inflammation and improved hearing with IL-1 blockade in MWS patients [11, 16–20]. MWS treatment options include anakinra [17], a short acting IL-1 receptor antagonist and canakinumab, a fully human monoclonal antibody providing selective and prolonged IL-1β blockade [21] and rilonacept, an IL-1 trap fusion protein [16].

Early detection of imminent hearing loss is crucial, yet challenging. Traditionally, pediatric and adult screening audiograms determine individual hearing thresholds at the frequencies 0.5, 1.0, 2, and 3 or 4 kHz reflecting those frequencies most relevant for speech discrimination. Hearing thresholds at each frequency are determined, and averaged in a single value, the so-called 4 pure tone average (4PTA: 0.5, 1, 2, and 4 kHz). This commonly used approach has significant limitations for the early detection of hearing loss in MWS, since the frequencies affected earliest are above the test range and therefore not included in the evaluation. However, early detection of imminent hearing loss and immediate initiation of targeted therapy may prevent progression to deafness in children and adults with MWS. Thus, a tailored assessment tool for detection of early hearing loss in MWS is urgently needed.

Therefore, the aims of the study were 1) to characterize the distinct pattern of hearing loss at diagnosis of MWS, 2) to modify the established standard 4PTA assessment tool to the hearing loss characteristics of MWS patients and assess its sensitivity in detecting hearing loss and 3) to determine risk factors associated with hearing loss in MWS and the effects of IL-1-inhibition.

## Methods

A single-center cohort study of consecutive patients diagnosed with MWS between 4/2004 and 12/2007 was performed. Pediatric and adult patients had to have a clinical diagnosis of MWS and genetic confirmation of a *NRLP3* mutation. The clinical diagnosis was based on the presence of MWS typical features of fever, non-purulent conjunctivitis, urticaria-like rash, sensorineural hearing loss, arthralgia/arthritis, fatigue coupled with raised inflammatory markers. Mutations were determined as previously described [22]. Exclusion criteria were 1) significant medical conditions impacting on hearing other than MWS and 2) age <3 years at diagnosis. Informed consent was obtained from all patients for the DNA sequence analysis of their *NLRP3* gene. The study was approved by the local ethics committee (Clinical Ethics Committee at the University Hospital Tuebingen, REB No 326/2007B01).

### Clinical features and disease activity

All MWS patients were diagnosed and followed according to a standardized protocol by an interdisciplinary tertiary care team. Demographic information included age at diagnosis, gender, ethnicity and type of *NLRP3* gene mutation. Clinical characteristics were documented as previously reported [23]. Review of systems captured constitutional symptoms of fever (pattern and duration) and fatigue. Organ specific clinical characteristics including headache, ocular symptoms of conjunctivitis, uveitis, papilledema and other eye symptoms, sensorineural hearing loss, oral ulcers, abdominal pain, arthralgia, arthritis and myalgia and skin symptoms including erythematous and cold-induced rash were collected. Proteinuria, hematuria and renal failure requiring organ replacement therapy were recorded. Inflammatory markers including C-reactive protein (CRP) and serum amyloid A (SAA) were determined in all patients. Disease activity was captured in the previously reported MWS-Disease Activity Score (MWS-DAS) [24]. MWS-DAS captures active MWS in 10 domains, nine of which reflect organ involvement of MWS including fever, headache, eye involvement, hearing impairment, oral ulcers, abdominal pain, renal disease, musculoskeletal disease, and rash in addition to the Patient Global Assessment Score. The MWS-DAS attributes 0, 1, or 2 points to each level of disease activity. Two points are given for severe symptoms, one point for mild symptoms, and zero points for the absence of symptoms in a domain. As previously determined, a MWS-DAS score of <10 points reflects overall mild MWS disease, whereas a score >10 points is an indicator of severe MWS disease activity.

### Hearing assessment

All patients had serial comprehensive otolaryngologic assessments. Pure-tone audiometry was serially performed for each ear separately using conventional, play or behavioral audiograms. All adult patients were additionally tested using speech audiometry, caloric vestibular stimulation and tinnitus handicap questioning. The audiograms of members of the same family were compared in order to capture the family-specific risk of progressive hearing loss. The audiologic examination included air and bone conduction threshold for pure tone frequencies of 0.125, 0.25, 0.5, 1, 2, 4, 6 and 8 kHz and middle ear functional testing by impedance audiometry. Hearing threshold is defined as the dB value at which sound is perceived at a given frequency. In order to account for normal age-related hearing loss, measured age-adjusted hearing thresholds were calculated (Additional file 1: Table S1).

### Hearing assessment tools

#### Pure Tone Average – 4PTA

Pure tone thresholds at 0.5, 1, 2 and 4 kHz were measured at each time point for each ear. The thresholds were summed and divided by 4 giving each frequency equal weight in the score. An average was calculated for each ear and examination time point (4PTA). Values were considered abnormal if they differed by ≥ 10 dB from normative hearing threshold levels (Additional file 1: Table S1) established from large groups of individuals matched in respect to age and frequency [25]. Comparison to normative data has been used in rare disease populations before [26]. Because of very minor gender differences especially in the younger age groups, male and female data were combined.

#### High frequency pure tone average – HF-PTA

High frequency pure tone average was determined by measuring pure tone thresholds at 6 and 8 kHz and averaging these thresholds giving each frequency equal weight in the score (HF-PTA). Values were considered abnormal if they differed by ≥ 10 dB from normative hearing threshold levels (Additional file 2: Table S2) established from large groups of individuals matched with respect to age and frequency [25]. Because of very minor genders differences especially in the younger age groups, male and female control data were combined.

#### WHO functional disability associated with hearing loss

**Fig. 1:**
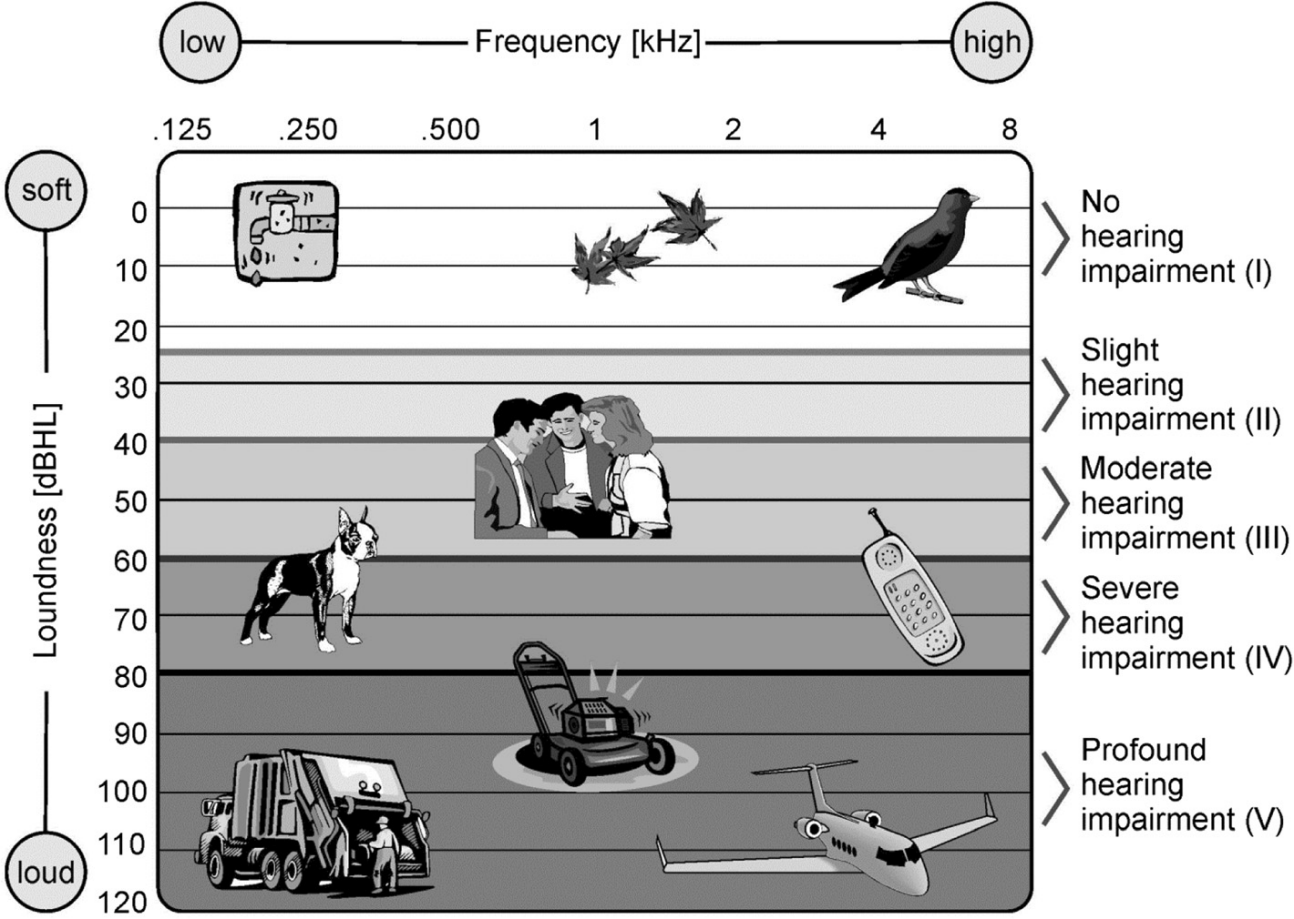
World Health Organization (WHO) definition of functional disability associated with hearing loss and frequencies of daily noises. The graph depicts common sources of noises including their frequencies and loudness. The WHO definition defines five severity grades of hearing impairment based on the 4PTA of the patient’s better ear. 4PTA captures the most relevant frequencies for speech discrimination at 0.5, 1, 2, and 4 kHz. Hearing impairment grades II to IV are considered disabling. Profound hearing impairment constitutes deafness.

Functional disability associated with hearing loss was evaluated according to the World Health Organization (WHO) (Fig. 1). WHO grades of hearing impairment in a patient are based on the 4PTA of the patient’s better ear. They include: grade 0: no hearing impairment (4PTA ≤ 25 dB), grade I: slight impairment (4PTA 26–40 dB), grade II: moderate impairment (4PTA children 31–60 dB; adults 41–60 dB), grade III: severe impairment (4PTA 61–80 dB) and grade IV profound hearing impairment, (4PTA ≥81db) (Fig. 1). Hearing impairment grades II to IV are considered disabling. Profound hearing impairment constitutes deafness. Functional disability levels were determined at baseline and at last follow-up.

#### Anti-IL-1 treatment

##### Anakinra therapy

Patients were treated with anakinra (Kineret; Amgen, Cambridge, UK) at dose of 100 mg/day for  ≥40 kg body weight or 1–2 mg/kg/day in patients <40 kg body weight. In children with persistent disease activity, the anakinra dose was stepwise increased to a maximum of 8 mg/kg/day. Anakinra was self-administered by subcutaneous injection once daily.

##### Canakinumab therapy

Patients received canakinumab subcutaneously 150 mg or 2 mg/kg for body weight <40 kg every 8 weeks. In case of residual symptoms, patients were maintained on a more intense dosing regimen (increase dose up to 600 mg s.c. or 8 mg/kg s.c and/or increase of dosing frequency).

#### Outcome

##### Primary outcome

Hearing loss was defined as increased threshold of ≥10 dB for any of the age-adjusted pure tone average for the frequencies 0.5, 1, 2 and 4 kHz (4-PTA) and the two high frequencies 6 and 8 kHz (HF-PTA).

##### Secondary outcomes

1) Improved or stable hearing was defined as a) decrease in hearing threshold by ≥ 20 dB in one frequency or by ≥10 dB in two, or more consecutive frequencies for improved hearing or b) absence of worsening of hearing at last follow-up. 2) Worsening of hearing was defined as increase in hearing threshold by ≥20 dB in one frequency, or by ≥10 dB in two or more consecutive frequencies, in accordance with definitions used in previous studies [15].

### Statistical analysis

Descriptive statistics were performed; results were reported as frequencies with percentages, means with standard deviations, or medians with ranges. Univariate parametric analyses utilized Fisher’s exact and chi-square analyses for categorical data and students T-test for continuous variables. Non-parametric tests were performed where appropriate. Paired univariable analyses were conducted to determine treatment responses. Repeated measures linear regression analysis was performed for comparative and predictive modeling of hearing thresholds to account for paired ears within patients. All tests considered *p* <0.05 as statistically significant. Statistical analyses were performed using SAS statistical software for Windows Version 9.3 (SAS Institute, Cary, North Carolina, USA).

## Results

A total of 23 patients with clinically and genetically confirmed MWS were included in the study; 65 % were females. The median age at diagnosis was 18 years with a range from 3–72 years. Fifty-two percent were children age <18 years. Three *NLRP3* mutations were found in this cohort: 61 % had the c.937G>A (p.Glu313Lys) formerly known as E311K mutation, 22 % the c.1049C>T (p.Thr350Met), formerly known as T348M and 17 % the c.598G>A (p.Val200Met), formerly known as V198M mutation (Table 1).

**Table 1:** Demographic, clinical and laboratory findings and characteristic patterns of hearing loss in patients with Muckle-Wells syndrome (MWS)

	Patients
	N = 23
Demographics	
Median age at diagnosis of MWS [years] (range)	18 (3–72)
Children ≤18 years at diagnosis (%)	12 (52)
Females (%)	15 (65)
NLRP3 mutation	
• c.937G>A (p.Glu313Lys) (%)	14 (61)
• c.1049C>T (p.Thr350Met) (%)	5 (22)
• c.598G>A (p.Val200Met) (%)	4 (17)
Overall disease activity	
Median MWS-DAS at baseline (range)	8 (6–16)
Laboratory markers	
Mean SAA at baseline in mg/l (std), (nl* <1)	6.87 (1.43)
Mean CRP at baseline in mg/l (std), (nl* <0.05)	0.22 (0.17)
Treatment	
IL-1 Inhibition (%)	23/23 (100)
Anakinra (%)	10 (43)
Canakinumab (%)	13 (57)

*nl: normal value

### Clinical features, disease activity and treatment

All patients had active disease at diagnosis reflected in a median MWS-DAS of 8. Most commonly seen clinical features were arthralgia (96 %), eye involvement (91 %) and headache (83 %). Clinical hearing loss was reported by 91 % of patients. Other features included oral ulcers (78 %), rash (65 %) and myalgia (52 %). At baseline, the mean SAA level was 68.7 mg/dl, mean CRP level 2.2 mg/dl (Table 1). Anakinra cohort: 10 patients (43 %) received anakinra treatment; these were three males and seven females. The median age at diagnosis was 17 years with a range from 3 to 44 years. The most common mutation was the c.937G>A (p.Glu313Lys) mutation present in 5 (50 %) patients; 3 (30 %) had the c.1049C>T (p.Thr350Met) and 2 (20 %) the c.598G>A (p.Val200Met) mutation. Canakinumab cohort: 13 patients (57 %), 5 males and 8 females, were treated with canakinumab. The median age at diagnosis was 34 years with a range between 3 and 72 years. Three different *NLRP3* mutations were found: c.937G>A (p.Glu313Lys) in nine and c.1049C>T (p.Thr350Met) and c.598G>A (p.Val200Met) in two respectively.

### Hearing assessment

**Fig. 2:**
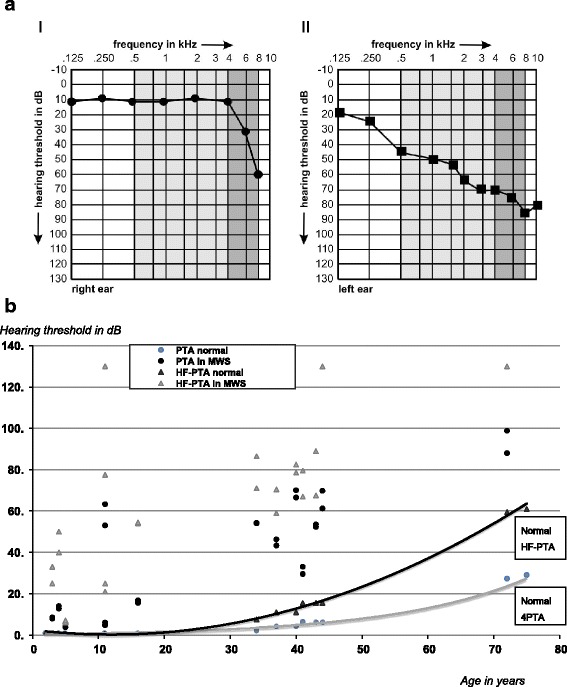
Variation of hearing thresholds across ages and frequencies in patients with Muckle-Wells syndrome (MWS). **a** Comparison of hearing threshold within a family with MWS. Audiogram of a 5 year-old child with MWS (**a-1**): Standard hearing assessment in the 4PTA range including 0.5, 1, 2, and 4 kHz was normal (*light gray area*). In contrast the proposed HF-PTA captured a dramatic early high frequency hearing loss with increased hearing thresholds at 6 and 8 kHz (*dark grey area*). The child’s 37-year old father reported hearing impairment. The corresponding audiogram (**a-11**) revealed abnormally increased hearing thresholds across all frequencies (*light and dark grey areas*). In the father advanced MWS associated hearing loss is captured not only by HF-PTA but also by standard 4PTA. **b** Comparison of hearing thresholds of standard 4 pure-tone-average (4PTA) and proposed high frequency pure-tone-average (HF-PTA) in children and adults with MWS and age-matched healthy controls across the age spectrum. Normal hearing thresholds captured in 4PTA and HF-PTA increase with age (4PTA *grey line*, HF-PTA *black line*). MWS patients of all ages have significantly higher 4PTAs (*black circles*) and HF-PTAs (*grey triangles*) even after adjusting for age-specific normal hearing thresholds

At baseline, 21/23 MWS patients (91 %) had sensorineural hearing loss by audiogram, correspondingly 42/46 hearing assessments (single ears) were abnormal. All 11 adults had abnormal hearing assessments (100 %, 22/22 ears), while only 10/12 children (83 %, 20/24 ears) had evidence of sensorineural hearing loss. Hearing thresholds at baseline varied significantly across ages and frequencies (Fig. 2). Comparison of hearing thresholds by frequency demonstrated significant differences between lower and higher frequencies, even after adjusting for age and gender (*p* <0.001). All patients had normal tympanic membrane mobility, normal middle ear pressure by impedance audiometry and normal caloric vestibular stimulation. Seven adult patients reported tinnitus.

**Fig. 3:**
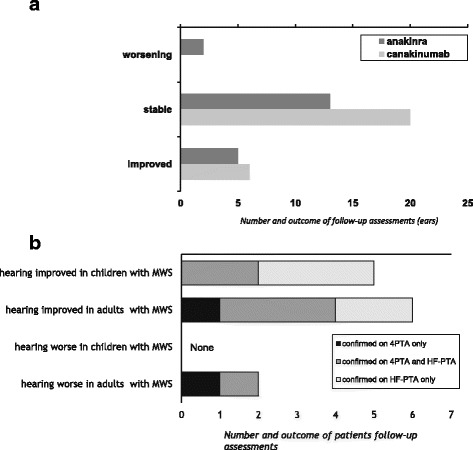
Effect of anti-IL-1 therapy on hearing assessments in patients with MWS. **a** Comparison of two different anti-IL-1 therapy regimens. A total of 11 assessments (ears) of 9 patients improved with anti-IL1 therapy; 4/9 were children, five were adults. None of the patients had worsening of the contralateral ear. Improvement occurred with both treatment regimens. All but two patients with hearing improvement were females. Overall, worsening was exclusively observed in adult patients, a male and a female. Both patients had received anakinra therapy. **b** Responsiveness to change: Comparison between 4PTA and HF-PTA follow-up assessments after exposure to anti-IL-1 therapy. 4PTA and HF-PTA was calculated in all MWS patients following anti-IL-1 therapy. Hearing improved in five children and six adults, most commonly captured by HF-PTA (10/11), either by HF-PTA only (5/10, *light grey bars*) or also by the standard 4PTA (5/10, *medium grey bars*). Hence, a total of 91 % of improved assessments were detected by HF-PTA, while just in one patient improvement was detected by standard 4PTA only. Worsening was not documented in children, but deteriorated in two adults. In both, this was detected by standard 4PTA, in one also in the HF-PTA

### 4PTA assessments

At baseline, 34/46 (74 %) MWS hearing assessments demonstrated an abnormal 4PTA, including 22/22 adult patient hearing assessments (100 %) and 12/24 (50 %) children ears. The median baseline 4PTA was 28 (range 0–72); at last follow-up, the median 4PTA was 26 (range 0–56) (Table 2).

**Table 2:** Audiologic findings in a cohort of pediatric and adult MWS patients at baseline and following treatment with anti-IL-1-therapy utilizing the standard 4PTA and the proposed high frequency (HF-PTA) instrument

	Patients N = 23
	Assessments (ears) N = 46
Hearing loss at baseline	
Patients with clinical hearing loss (%)	21/23 (91)
Ears (assessments) with hearing loss (total) (%)	42/46 (91)
Adults with hearing loss (%)	11/11 (100)
• Abnormal assessments (adults) (%)	22/22 (100)
Children with hearing loss (%)	10/12 (83)
•Abnormal assessments (children) (%)	20/24 (83)
Hearing thresholds at baseline	
4-frequency pure tone average (4PTA), median (range)*	28 (0–72)
Number of abnormal 4PTA assessments (%)*	34/46 (74)
•Adult assessments with abnormal 4PTA (%)	22/22 (100)
•Pediatric assessments with abnormal 4PTA (%)	12/24 (50)
High Frequency HF-PTA median (range)*	45 (0–114)
Abnormal HF-PTA hearing assessments (%)	42/46 (91)
•Adult assessments with abnormal HF-PTA (%)	22/22 (100)
•Pediatric assessments with abnormal HF-PTA (%)	20/24 (83)
Hearing thresholds at last follow-up	
4 Pure Tone Average (4PTA); median (range)*	26 (0–56)
Abnormal 4PTA assessments (%)	34/46 (74)
High Frequency HF-PTA; median (range)*	43 (0–114)
Abnormal HF-PTA assessments (%)	42/46 (83)
Sensitivity of hearing assessments	
Detection of clinical hearing loss by 4PTA	81 %
Detection of clinical hearing loss by HF-PTA	100 %

* Each patient is represented twice, since each ear had a separate hearing assessment; all values are adjusted for age (Additional files 1 and 2: Tables S1 and S2)

### HF-PTA

At baseline, 42 MWS patient assessments demonstrated an abnormal HF-PTA (91%). An abnormal HF-PTA was found in 22/22 adult assessments (100 %) and in 20/24 (83 %) in children. The median baseline HF-PTA was 45 (range 0–114); at last follow-up, the median HF-PTA was 43 (range 0–114) (Table 2).

### Sensitivity of PTA assessments

The sensitivity of the 4PTA to detect clinical hearing loss was 81 %. The 4PTA assessment exclusively missed children with early severe hearing loss affecting primarily high frequencies. The HF-PTA testing identified 100 % of patients.

### WHO functional disability associated with hearing loss

At baseline, 8 patients – all children – had no hearing impairment (WHO grade 0), two patients, both adults, showed slight impairment (WHO grade I), eight – four children and four adults – had moderate (grade II), four had severe hearing impairment (WHO grade III), all were adults, and one adult had profound hearing impairment (WHO grade IV).

At last follow-up, 9 patients – all children – presented with no hearing impairment (WHO grade 0), one adult showed slight (WHO grade I), nine patients, six adults and three children, had moderate (grade II), three patients, all adults (WHO grade III), and one adult still had profound hearing impairment (WHO grade IV). So one child reversed hearing loss during treatment.

### Risk factors for hearing loss in MWS

Female gender (*p* = 0.008), earlier age at diagnosis (*p* <0.001), and affection of high compared to low frequencies (*p* = <0.001) were found to be significantly associated with hearing loss in a repeated measures linear regression model to account for paired ears within patients. Young age was primarily associated with high frequency hearing loss, while older patients had evidence of abnormal hearing thresholds across all frequencies. Female gender was an independent risk factor for hearing loss in MWS.

### Effect of anti-IL-1 therapy on hearing loss

A total of 44/46 (96 %) patient ears had improved or stable hearing assessments as defined above when treated with anti-IL1 therapy. Improvement was documented in 11 ears, six of which were treated with canakinumab and five with anakinra. Stable hearing was documented for 33 ears, 20 of which were treated with canakinumab and 13 with anakinra. Only two ears worsened significantly; these include one of an 18-year-old male and one of a 37-year-old female, both treated with anakinra. The contralateral ears for the two patients remained stable (Fig. 3).

## Discussion

This is the first study to systematically analyze the spectrum of hearing loss in a large prospective cohort of MWS patients across all ages. A key finding is that early inner ear inflammation in MWS primarily affects high frequencies above 4 kHz and remains undetected when using standard assessment approaches (4PTA). Therefore, optimized testing for early hearing loss in MWS has to include high frequency hearing thresholds of 6 and 8 kHz (HF-PTA) in order to significantly increase the sensitivity of the hearing assessment and to allow for the recognition of early MWS associated hearing loss due to inflammatory injury. This is particularly important in children, since early on in life MWS solely affects high frequencies. In fact, the study determined that earlier age at diagnosis was significantly associated with the development of hearing loss. Rapid onset of therapy may control the inflammation thus greatly reducing the risk of inner ear damage and deafness in MWS [24]. The study confirmed that anti-IL-1 therapy is effective in stabilizing or even improving sensorineural hearing loss, particularly in younger patients. Improved hearing is best confirmed when utilizing the optimized assessment tool HF-PTA in addition to standard testing. Reversibility of hearing loss follows the MWS pattern and is most commonly seen in high frequencies. The study also highlighted that female MWS patients of all ages are at significantly higher risk for hearing loss and deafness compared to males. Importantly, females of all ages had a good response to anti-IL-1 therapy.

Onset and progression of hearing loss in MWS has a distinct pattern (Fig. 2a). The inflammatory attack primarily affects high frequencies. A similar hearing loss pattern has been described in other inflammatory diseases including rheumatoid arthritis [12], metabolic diseases including diabetes [13, 27] and thyroid disease [28] and exposure to ototoxic drugs [29]. Hearing thresholds in the high frequencies of the pure tone audiogram are particularly relevant for speech discrimination and communication, especially in a noisy environment [30]. Affected MWS patients report impaired hearing; however standard evaluation may remain normal. Detection of early hearing loss in MWS mandates testing of high frequency hearing thresholds. Early inner ear injury may remain unnoticed when using the current testing routine (4PTA). The proposed HF-PTA has a higher sensitivity for early disease and should therefore be considered in all ages, but particularly in younger MWS patients. An extended hearing threshold testing (0.5-8 kHz) is increasingly used for noise induced hearing loss [31] and when monitoring for potential ototoxicity with antibiotics and chemotherapeutic agents [29]. Overall, MWS patients require comprehensive otolaryngologic assessments. Serial pure tone thresholds including air- and bone conduction thresholds for frequencies of 0.125, 0.25, 0.5, 1, 2, 4, 6 and 8 kHz should be determined by pure-tone audiometry using conventional, play or behavioral audiograms. Speech audiometry, caloric vestibular stimulation, tinnitus assessments and middle ear functional testing by impedance audiometry should be included.

Prevention of deafness as a result of inner ear inflammation in children and adults is a key task when treating patients with MWS. Anti-IL-1 therapy was effective in stabilizing progression of hearing loss in this cohort, similarly to what was reported by others caring for patients with CAPS [15, 32–34]. In our study, reversibility of inner ear disease was found to follow a characteristic pattern with early improvement in the high frequencies. Change was best detected by HF-PTA and may be missed when testing standard frequencies only. Overall, it appears that there is a window of opportunity at the time of primary involvement of high frequencies. Correspondingly, younger patients were most likely to respond to treatment. The window may vary between different *NLRP3* mutations [11]. Overall, younger age was clearly identified to be a risk factor associated with sensorineural hearing loss. Females with MWS appear to be at higher risk for hearing loss at all ages, correspondingly they were previously found to have overall more severe disease as captured by the MWS disease activity score (MWS-DAS) [24]. Reasons for this observation are unknown. The unbalanced gender ratio in this study only reflects the gender of the consecutive patients included into this study at time of inclusion. As the gender distribution in other larger cohorts of CAPS patients is roughly balanced (own complete CAPS cohort: 55 % males, β-confident registry: 54 % females) in CAPS no gender preference can be observed. Also, type of mutation is associated with different degrees of hearing loss, as described previously [11]. Interestingly, in this study all but two patients with evidence of hearing improvement after anti-IL-1 therapy were females.

There are several imitations to the study. The number of patients tested is small, only 23 children and adults were included. However, MWS is a very rare condition. A comprehensive, standardized assessment and therapy protocol was applied prospectively to all patients allowing for the capture of detailed clinical and audiological data at baseline and following therapy. The age spectrum was wide with relatively few patients per age group threatening the generalizability of the results. However, the clinical spectrum of CAPS is also wide and varies further between *NLRP3* gene mutation types regarding impact on hearing [24]. The study included three different mutations and almost balanced numbers of children and adults. The study did not include additional objective measures of hearing such as otoacoustic emissions, brainstem evoked response audiometry and gadolinium-enhanced magnetic resonance imaging (MRI) studies. However, these tests have limited utility as generalizable screening and monitoring tools for centers worldwide. In addition, MRI studies of the inner ear in children commonly require anesthesia.

## Conclusions

Diagnostic evaluation and monitoring of hearing abilities in patients with MWS mandates a comprehensive otolaryngologic approach. The addition of HF-PTA to the standard 4PTA pure tone threshold testing allows for early recognition of imminent hearing loss and should prompt the rapid start of anti-IL-1 therapy for prevention of damage in particular in the high risk MWS populations.

## Additional files

Additional file 1: Table S1. Median level of normal hearing across age groups and frequencies (modified from Spoor [25]). (DOCX 24 kb) Additional file 2: Table S2. Age-group specific median limits of normal hearing (calculated from Spoor 1967 [25], for the 4-frequency pure tone average (4PTA_0.5-4kHz_) and the proposed high-frequency pure tone average (HF-PTA_6,8kHz_). Because of very minor gender differences, especially in the younger age groups, male and female data were combined. (DOC 51 kb)
