# Supplementary material for: Early detection of sensorineural hearing loss in Muckle-Wells-syndrome
Source: Pediatr Rheumatol Online J. 2015 Nov 4;13:43. doi: 10.1186/s12969-015-0041-9 (PMC4632838; doi:10.1186/s12969-015-0041-9)
Supplement: Additional file 1: Table S1. — Median level of normal hearing across age groups and frequencies (modified from Spoor [25]). (DOCX 24 kb) [file 12969_2015_41_MOESM1_ESM.docx]

Additional file 1: Table S1: Median level of normal hearing across age groups and frequencies (modified from Spoor 1967 [25]).

| Age in years | **Median level of normal (MLN) hearing [dB HL** | | | | | |
| --- | --- | --- | --- | --- | --- | --- |
|  | 0.5 kHz | 1 kHz | 2 kHz | 4 kHz | 6 kHz | 8 kHz |
| 10 | 1 | 1 | 1 | 1 | 1 | 1 |
| 15 | 1 | 1 | 1 | 1 | 1 | 1 |
| 20 | 1 | 1 | 1 | 1 | 1 | 1 |
| 25 | 1 | 1 | 1 | 1 | 1 | 1 |
| 30 | 1 | 1 | 1 | 2 | 3 | 2 |
| 35 | 1 | 1 | 2 | 4 | 6 | 5 |
| 40 | 2 | 2 | 4 | 8 | 10 | 8 |
| 45 | 3 | 3 | 5 | 10 | 14 | 13 |
| 50 | 4 | 4 | 8 | 15 | 18 | 17 |
| 55 | 6 | 6 | 10 | 19 | 23 | 23 |
| 60 | 8 | 8 | 14 | 24 | 29 | 30 |
| 65 | 10 | 11 | 15 | 29 | 35 | 38 |
| 70 | 13 | 13 | 21 | 35 | 42 | 47 |
| 75 | 16 | 17 | 26 | 41 | 49 | 57 |

dB decibel, HL hearing level
