# Supplementary material for: Early detection of sensorineural hearing loss in Muckle-Wells-syndrome
Source: Pediatr Rheumatol Online J. 2015 Nov 4;13:43. doi: 10.1186/s12969-015-0041-9 (PMC4632838; doi:10.1186/s12969-015-0041-9)
Supplement: Additional file 2: Table S2. — Age-group specific median limits of normal hearing (calculated from Spoor 1967 [25], for the 4-frequency pure tone average (4PTA0.5-4kHz) and the proposed high-frequency pure tone average (HF-PTA6,8kHz). Because of very minor gender differences, especially in the younger age groups, male and female data were combined. (DOC 51 kb) [file 12969_2015_41_MOESM2_ESM.doc]

Additional file 2: Table S2: Age-group specific median limits of normal hearing (calculated from Spoor 1967 [25], for the 4-frequency pure tone average (4PTA_0.5-4kHz_) and the proposed high-frequency pure tone average (HF-PTA_6,8kHz_). Because of very minor gender differences, especially in the younger age groups, male and female data were combined.

| Age in years | Pure tone audiometry averages | |
| --- | --- | --- |
|  | 4PTA_0.5-4kHz_ | HF-PTA_6,8kHz_ |
| 10 | 1 | 1 |
| 15 | 1 | 1 |
| 20 | 1 | 1 |
| 25 | 1 | 1 |
| 30 | 1 | 3 |
| 35 | 2 | 6 |
| 40 | 4 | 9 |
| 45 | 5 | 14 |
| 50 | 8 | 18 |
| 55 | 10 | 23 |
| 60 | 14 | 30 |
| 65 | 16 | 37 |
| 70 | 21 | 45 |
| 75 | 25 | 53 |
